# Low-temperature, vacuum-free deposition of N-DMBI-H enables high conductivities in organic semiconductors

**DOI:** 10.1039/d6ra05776d

**Published:** 2026-08-28

**Authors:** Federico Ferrari, Oliver Pokerznik, L. Jan Anton Koster

**Affiliations:** a Zernike Institute for Advanced Materials, University of Groningen Nijenborgh 3 Groningen 9747 AG The Netherlands l.j.a.koster@rug.nl

## Abstract

Doping is an essential process for controlling the electronic properties of organic semiconductors. While n-type doping has advanced significantly in recent years, processability remains a critical bottleneck: conventional methods rely on inert atmospheres or vacuum systems, increasing complexity and costs. To overcome these limitations, we developed a low-temperature, atmospheric-pressure vapor doping method using the commercially available dopant N-DMBI-H. Optimized for the prototypical fullerene derivative PCBM, this approach yields conductivities exceeding 1 S cm^−1^, exceeding those achieved by traditional bulk doping. Critically, the mild deposition conditions enable entirely ambient processing, from doping to encapsulation, with only minimal performance trade-offs. This simplification reduces pipeline complexity, processing costs, and environmental impact, paving the way for scalable fabrication of high-performance n-type organic films.

## Abstract 2

Deposition of n-type dopants for organic electronics often requires complex vacuum-based systems and moderate temperatures. Here, we show that N-DMBI-H, the most common n-type dopant, can be deposited in ambient air without any complex equipment.

## Graphical abstract

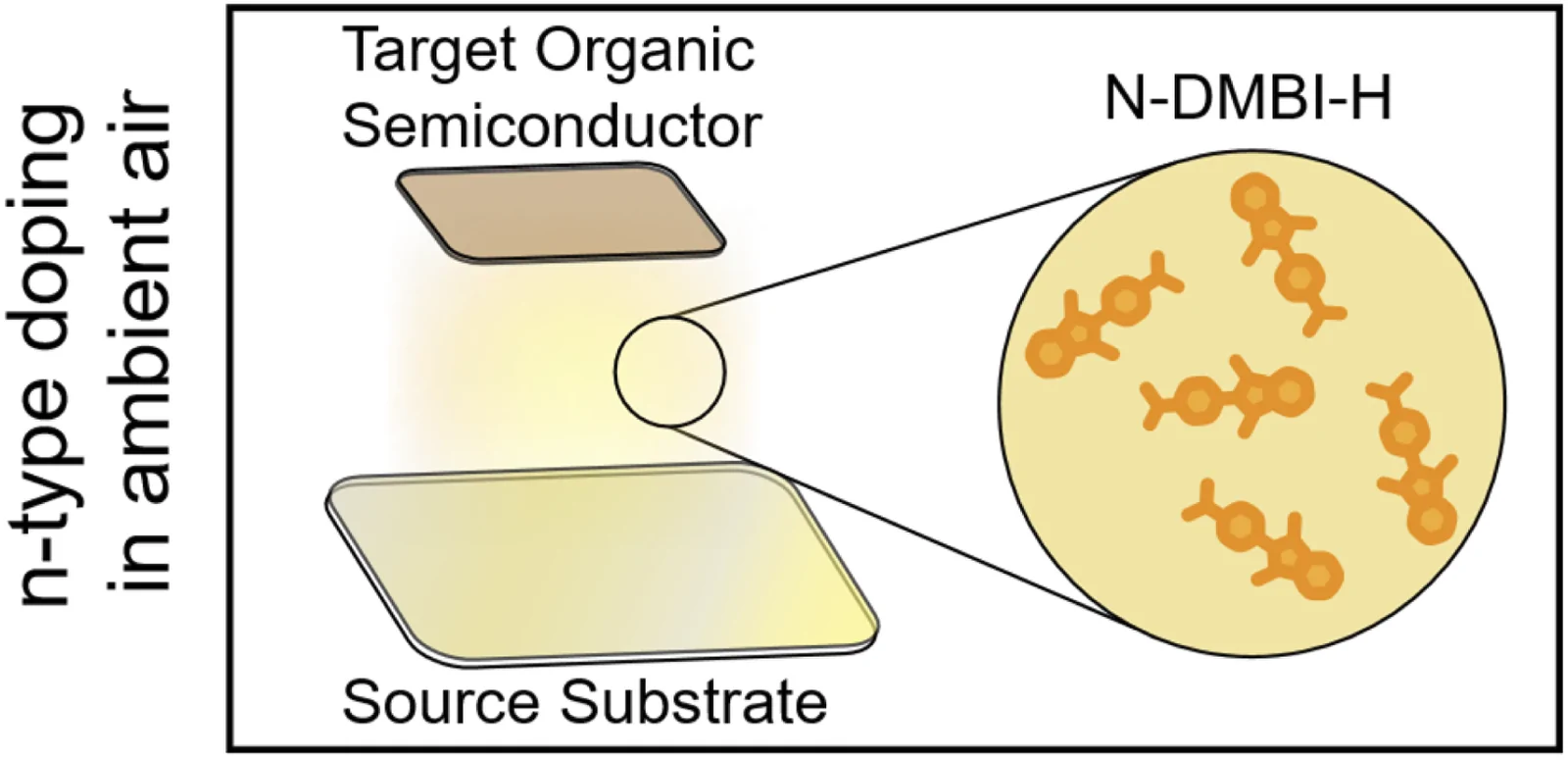

## Introduction

The large-scale fabrication of organic semiconductor (OSC)-based devices—such as organic light-emitting diodes (OLEDs)—relies heavily on vacuum thermal evaporation, a process that demands specialized equipment and drives up manufacturing costs.^1,2^ This economic barrier is compounded by the air instability of most n-doped OSCs, which necessitates additional encapsulation steps, further increasing production expenses.^2^

The versatile chemistry of OSCs allows their molecular structures to be tailored for solubility, enabling deposition *via* low-cost techniques like spin-coating or inkjet printing. Solution processing is widely adopted in academic research as an alternative to vacuum-based methods. However, this approach has severe drawbacks that prevent its adoption on a large scale.^1^ Many π-conjugated systems dissolve only in environmentally harmful solvents,^3^ and the choice of solvent, along with the deposition method, significantly affects the doped material's properties.^4,5^ Bulk doping, a common solution-processing method, involves pre-mixing the semiconductor and dopant in a controlled ratio before co-deposition. While conceptually simple and straightforward, this method suffers from poor reproducibility in practice. Variables such as mixing time are often not rigorously controlled, leading to significant variability in reported properties for nominally identical systems.

Sequential doping presents a potential workaround, wherein the dopant is introduced after semiconductor deposition. Common approaches include spin-casting the dopant onto the surface or immersing the OSC in a dopant-containing solution. Different variations, such as ion exchange,^6,7^ proton-coupled electron transfer,^8^ and inverse sequential doping,^9^ emerged as promising approaches but share a key limitation: the initial OSC layer must resist dissolution in subsequent processing solvents. Moreover, these wet-process methods exacerbate the environmental and reproducibility issues inherent to solution-based techniques.

Another method involves the sequential deposition of the dopant through vapor-phase deposition. This approach requires heating a solid organic compound to increase its vapor pressure and allow it to condense on the target substrate. However, the vapor pressure of these materials is far lower than atmospheric pressure, which inhibits significant evaporation rates under ambient conditions. For this reason, the technique requires high-vacuum conditions, with typical pressures ranging from 10^−2^ torr down to 10^−11^ torr.^10,11^ The use of vacuum also provides additional benefits: it minimizes unwanted side reactions by removing atmospheric reactants and suppresses convection, reducing heat loss and enabling uniform deposition. Furthermore, even under reduced pressure, heating the material is almost always necessary to achieve a practical evaporation rate.

The temperature required to achieve successful deposition depends on the material's sublimation enthalpy, the desired deposition rate, the chamber pressure, and the geometry of the experimental apparatus, but it must always remain below the degradation temperature of the material. Typical values range from 150 °C to 400 °C.^11–14^ While these systems allow precise control over morphology, they are inherently complex, limiting accessibility and requiring substantial investments (see SI Table S1).^15,16^ A notable alternative to vacuum-based vapor deposition is tetrakis(dimethylamino)ethylene (TDAE).^17–19^ As a liquid under ambient conditions, TDAE allows deposition at atmospheric pressure, eliminating the need for vacuum systems. However, this advantage is offset by its poor air stability, which necessitates expensive inert-atmosphere equipment during processing.

To address the limitation of air-stability, benzoimidazole-based dopant precursors have emerged as alternative.^20^ These compounds can react with semiconductor hosts *via* thermal, photochemical, or catalytic activation after their deposition. While primarily employed in solution processing, Wei *et al.* demonstrated vacuum co-evaporation of a methoxylated benzoimidazole salt with C_60_, achieving high conductivities (up to 5.5 S cm^−1^).^21^ However, in this study there are some drawbacks, the deposition relies on a vacuum system and the dopant gets activated during the deposition process requiring the handling and characterization of the sample to be carried out in a glove box.

In this work, we address these issues by developing a ambient-air vapor-phase doping process with the prototypical n-type dopant N-DMBI-H [(4-(1,3-dimethyl-2,3-dihydro-1*H*-benzoimidazol-2-yl)phenyl)dimethylamine]. This solvent-free, ambient processing method eliminates the need for specialized equipment for processing of n-doped organic semiconductors. By optimizing the process for the prototypical acceptor [6,6]-phenyl-C_61_-butyric acid methyl ester (PCBM), we achieve conductivities exceeding 1 S cm^−1^.

## Results and discussion

**Fig. 1:**
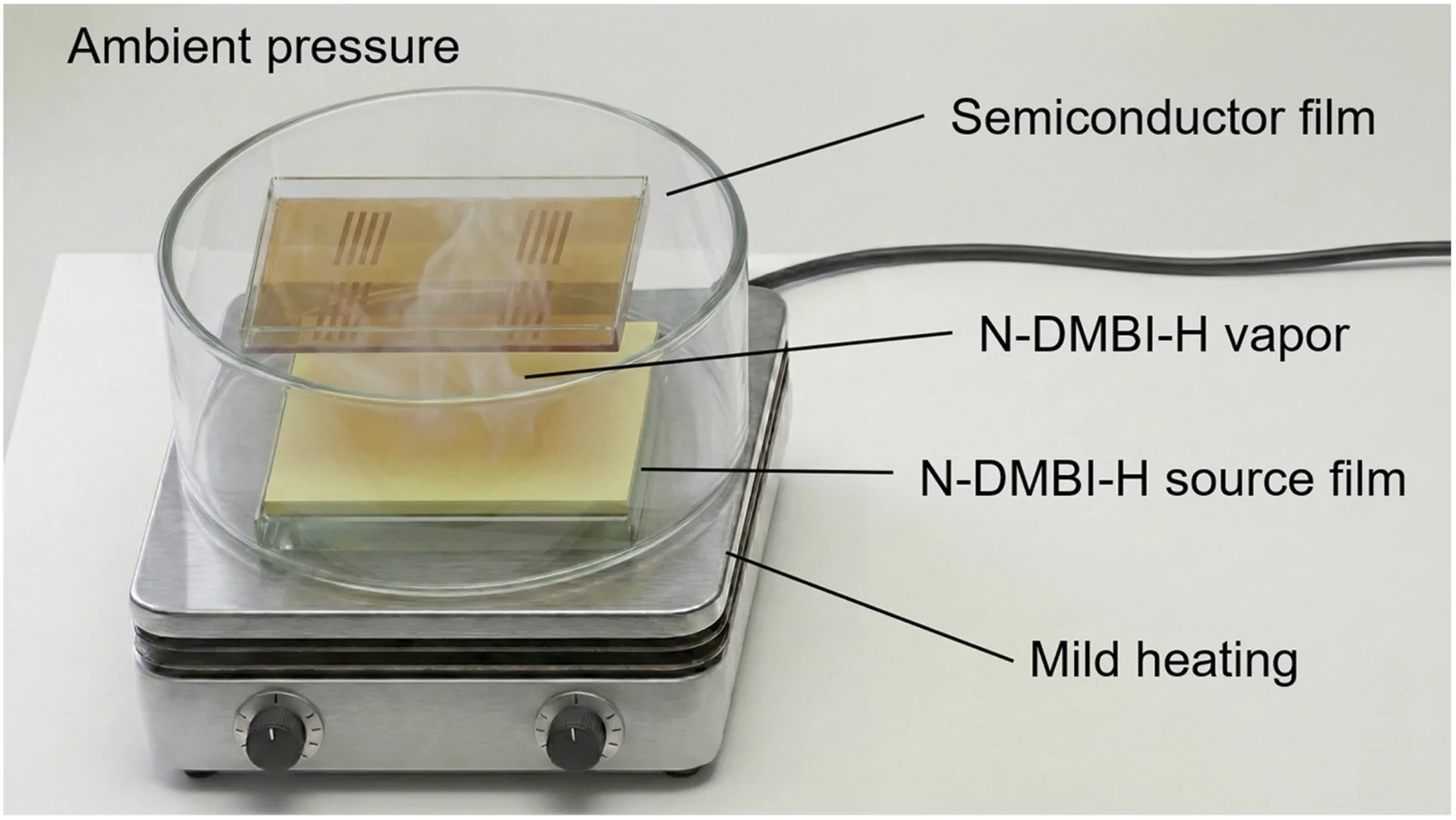
Schematic of the vapor doping process. An N-DMBI-H film is deposited on a glass substrate and mildly heated at 85 °C. The target semiconductor film is placed facing the dopant source at a specified distance. All is enclosed by a Petri dish.

The vapor doping process is illustrated schematically in Fig. 1 and described in detail in the Materials and methods section. In short, a N-DMBI-H source film is prepared *via* spin casting at low speed, the target semiconductor was placed facing the source film. All is placed on a hot plate and enclosed with a Petri dish. Crucially, the deposition is carried out in a glove box at a pressure of ∼1 atm at mild temperatures (<120 °C). After dopant deposition, the semiconductor film undergoes annealing for 1 hour. The efficacy of the doping procedure was evaluated by measuring the film's conductivity using the four-point probe method.

We selected PCBM as the semiconductor to test the method due to its commercial availability, relatively low cost (compared to other high-performance electron-transporting organic materials), and because its conductivities have been reported for a variety of dopants and processes.

**Fig. 2:**
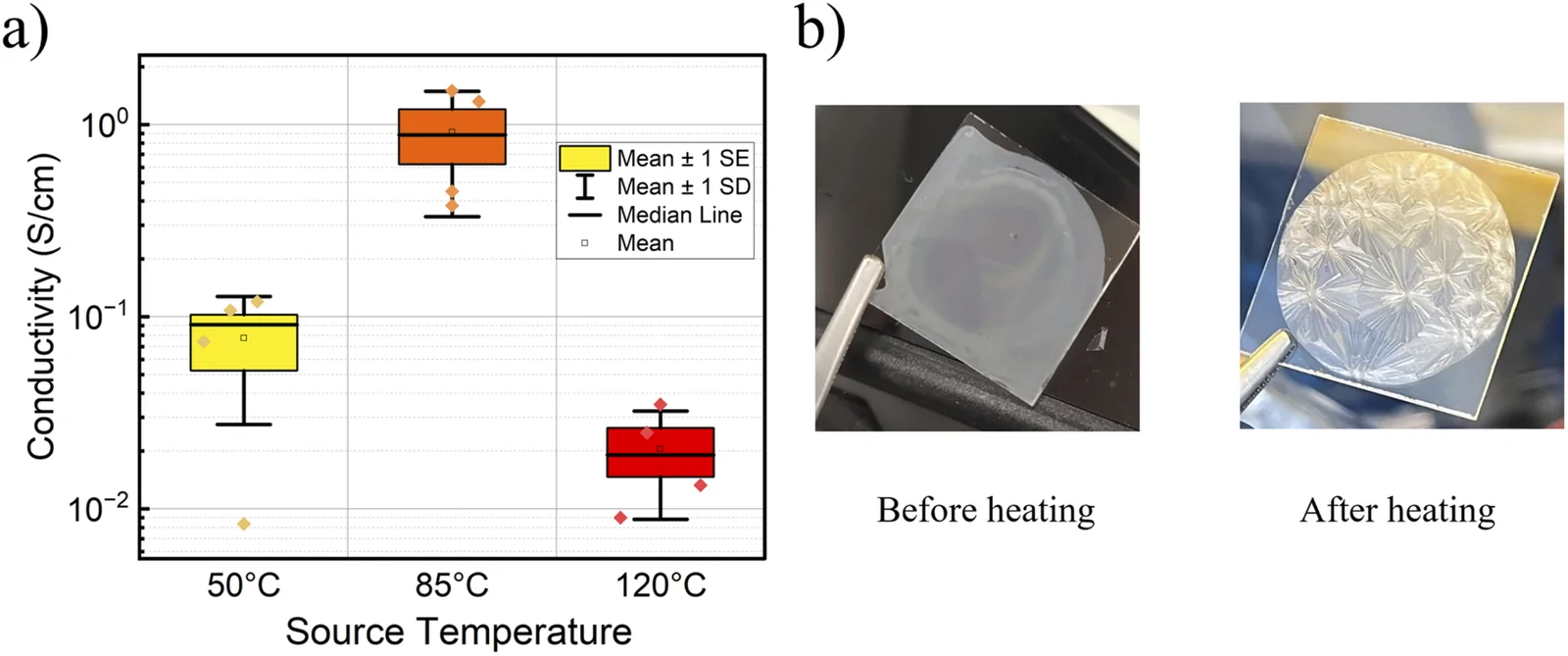
(a) Conductivities at room temperature obtained for vapor doped PCBM exposed for 25 minutes to a N-DMBI-H source heated at different temperatures; (b) a N-DMBI-H source before (left) and after (right) heating at 85 °C.

To establish a protocol for this process, we first investigated the effect of the source temperature as it strongly influences the vapor flux. The results show that doping is effective with a source temperature as low as 50 °C yielding an average conductivity of 0.08 S cm^−1^ (see Fig. 2a). This value increases to a remarkable 0.45 S cm^−1^ with a source temperature of 85 °C with a champion value exceeding 1 S cm^−1^. This is among the highest conductivities for such systems surpassing more advanced techniques such as inverse sequential doping by more than a factor of 2.^22^ Notably, at this temperature the N-DMBI-H film formed macroscopic crystals (see Fig. 2b). This observation aligns with the findings of Pallini *et al.*,^23^ who identified through differential scanning calorimetry that cold crystallization of N-DMBI-H happens between 50 °C and 75 °C. At 120 °C the conductivity drops to 0.02 S cm^−1^. The increase in conductivity when increasing the source temperature from 50 °C to 85 °C is likely due to a higher amount of dopant being deposited on the semiconductor whereas the subsequent decrease at 120 °C might be due to N-DMBI-H reacting before reaching the surface of the OSC film.

**Fig. 3:**
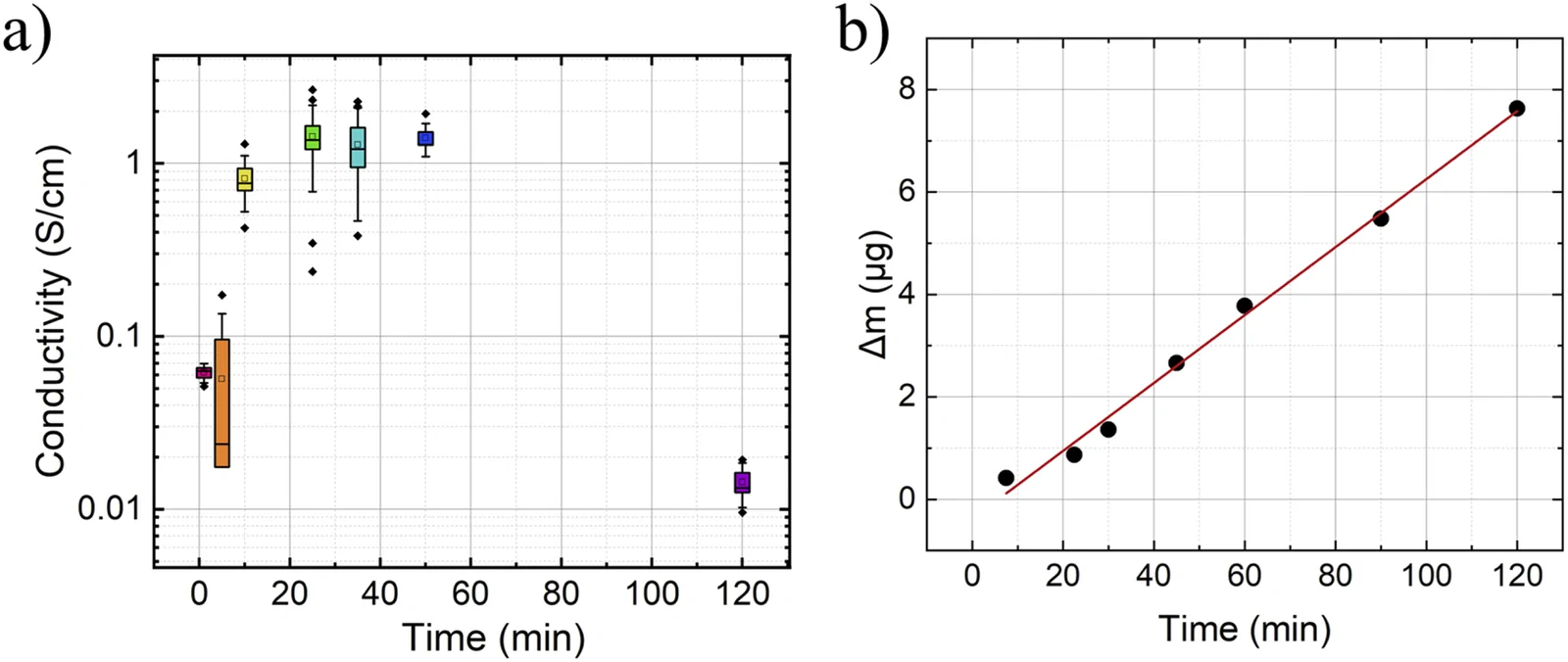
(a) Conductivity of PCBM films upon N-DMBI-H vapor exposure for different durations (source temperature: 85 °C); (b) mass variation of a quartz crystal upon exposure to NDMBI vapor for different exposure times, the linear fit yields a deposition rate of 0.043 µg min^−1^ cm^−2^.

We then optimized the effect of deposition time on the film conductivity. We were able to achieve an average conductivity above 1 S cm^−1^ with an exposure time between 25 and 50 minutes (see Fig. 3a). We investigated the film morphology using atomic force microscopy (AFM), which revealed that N-DMBI-H did not form a continuous film but rather aggregated into islands with dimensions of a few hundred nanometers (see SI Fig. S1). Therefore, to better quantify the deposited dopant, we measured the deposited mass using a quartz crystal microbalance (see Fig. 3b). We tracked the frequency shift of the crystal's resonant peak during deposition and used the Sauerbrey equation to convert it into mass variation. In Fig. 3b is shown the deposited mass over time, the deposition rate was determined to be approximately 43 ng min^−1^ cm^−2^ at a source-substrate distance of 11 mm and a source temperature of 85 °C. Since we used the same experimental conditions to deposit the dopant on top of the PCBM films we used this rate to estimate a dopant loading between 30% mol and 95% mol.

For comparison, we also tested the procedure using TDAE, another n-type dopant often evaporated without a vacuum chamber. However, the conductivities obtained with TDAE were significantly lower than those achieved with N-DMBI-H (see SI Fig. S2).

**Fig. 4:**
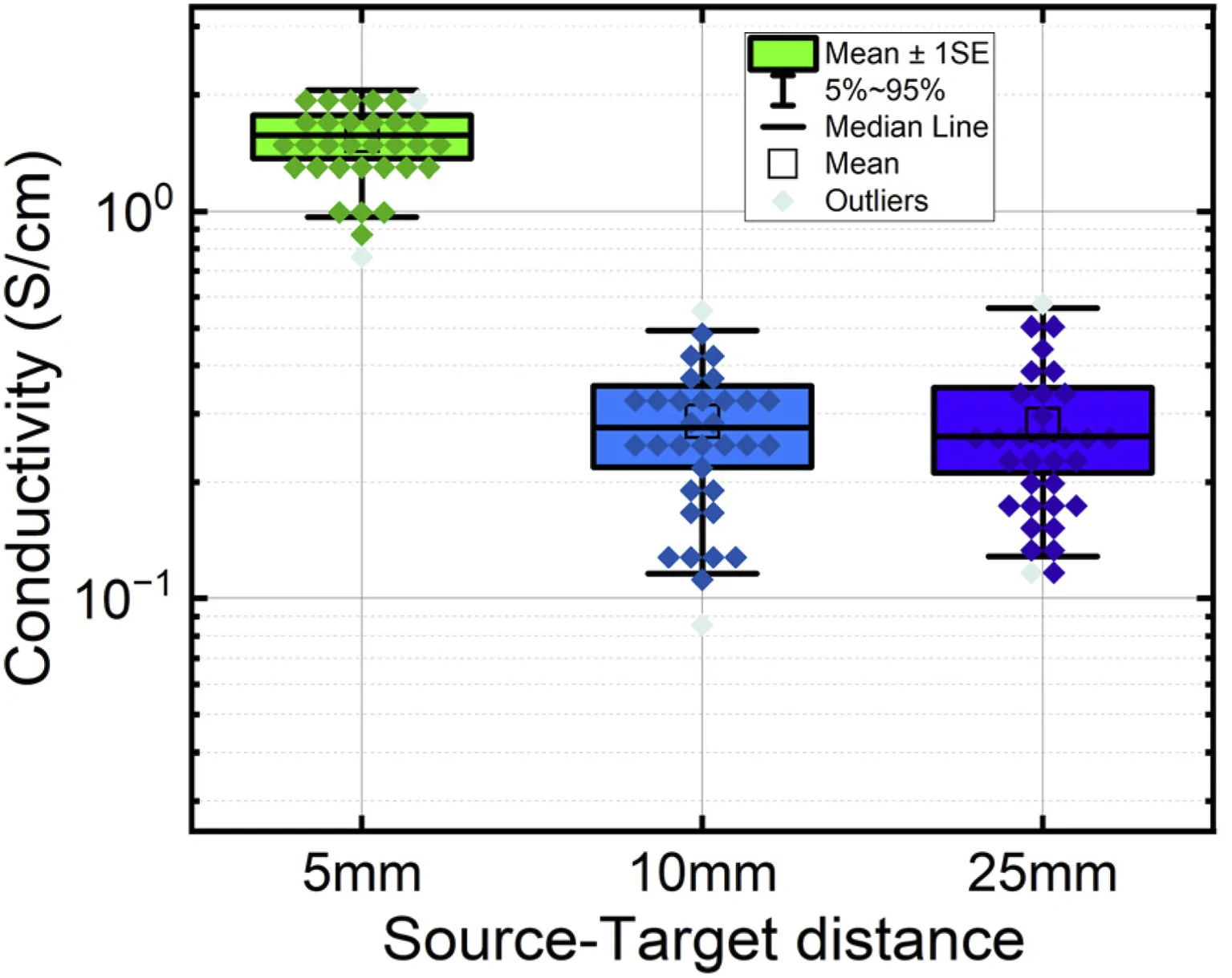
Conductivity of PCBM films upon N-DMBI-H vapor exposure for different source-target distances (source temperature: 85 °C).

Compared to bulk doping, vapor deposition yields a much higher variability with conductivities varying over 10-fold in the same sample. However, it does not fundamentally alter the transport of the films as can be observed from the similar relationship between the thermopower and the electrical conductivity (see SI Fig. S6). To optimize the deposition parameters for the reduction of variability we changed substrate geometry. We used a larger glass substrates (30 mm × 30 mm) with 11 pairs of gold electrodes (see SI Fig. S3). We found that decreasing the distance between the dopant source and the target film reduces significantly the variability of the process from a relative variance of ∼40% down to 22%. In addition, the shorter distance also provides higher conductivities reaching 2 S cm^−1^ rivaling with the highest conductivity reported for PCBM and obtained with the use of a organic superbase with limited air stability^24^ (Fig. 4).

It has been shown how good host-dopant solubility and a uniform dopant distribution are necessary in order to maximize conductivity and the doping efficiency.^25–27^ This ideal scenario is far from the conditions of our system right after the dopant deposition. In fact, in most of sequential doping approaches the dopant deposited solely at the interface with the semiconductor and then it penetrates throughout the bulk of the semiconductor usually *via* diffusion aided by thermal activation during an annealing step.

Therefore, we decided to investigate how the annealing temperature affects the conductivity of the semiconductor. A key challenge arises from the temperature dependence of both dopant diffusion and dopant activation. To disentangle these two effects, we hypothesized that they occur on different timescales. Therefore, we monitored the evolution of conductivity over the course of the annealing process.

**Fig. 5:**
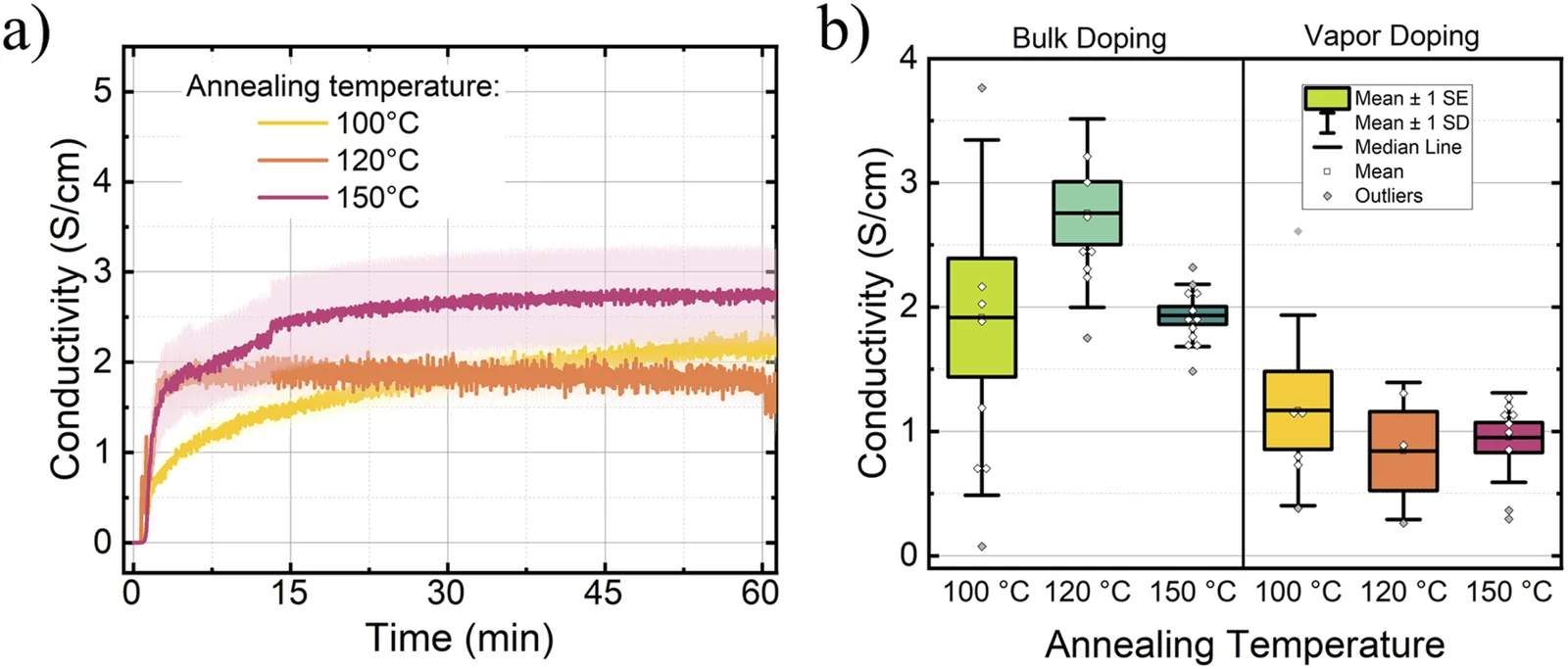
(a) Average trends in conductivity of multiple measurements (at least 11 from three different samples) during the annealing at three different temperatures for vapor doped PCBM. (b) Room temperature conductivities for bulk doped and vapor doped PCBM after annealing at different temperatures.

Measurements were conducted using a custom-built setup, where the sample was placed on a temperature-controlled stage while resistance was continuously recorded (see SI Fig. S4). The results, presented in Fig. 5a, show the average conductivity trends from multiple measurements (at least 11 measurements across three different samples) during annealing at three distinct temperatures.

The data reveal a rapid increase in conductivity in the first two minutes of the measurements corresponding to the stage reaching the setpoint temperature (see inset of Fig. 5a). During this phase, the N-DMBI-H on the PCBM surface activates and ionizes to N-DMBI^+^.^28^ In the following minutes there is a steady increase in conductivity due to the diffusion of the dopant into the bulk of the film. This gradual increase in conductivity is much more limited in the case of bulk doped samples (see SI Fig. S5) denoting how it is indeed an effect of thermally activated diffusion. Despite the different annealing temperatures, the final conductivity at room temperature is not significantly affected.

**Fig. 6:**
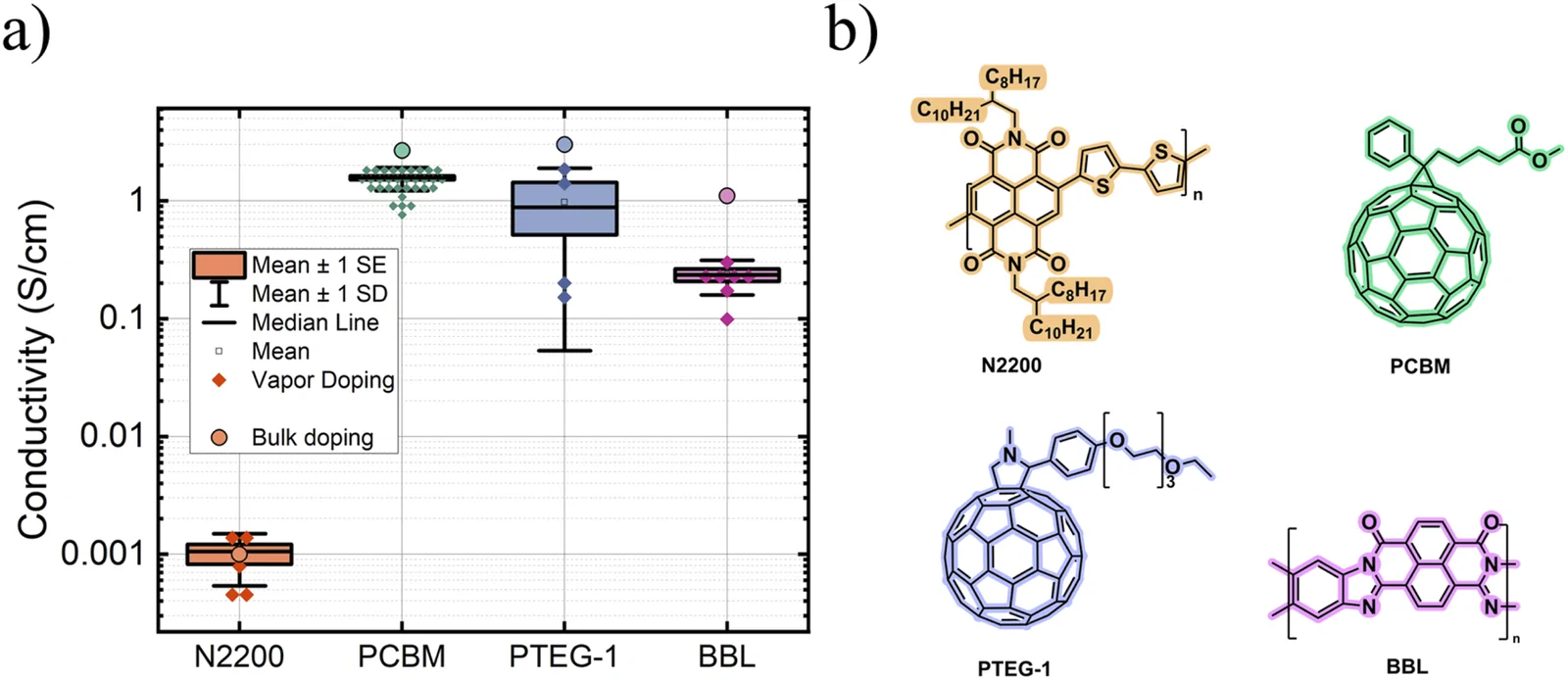
(a) Boxplot of the conductivities obtained *via* vapor doping for prototypical n-doped organic semiconductors: N2200, PCBM, PTEG-1, and BBL and (b) their respective chemical structure. The values reported for bulk doping are based on ref. 29 and 30 for N2200, ref. 33 for PCBM, ref. 25 and 31 for PTEG-1, and ref. 32 for BBL.

To demonstrate the versatility of our vapor doping method, we applied it to three additional, well-studied organic semiconductors and compared the results with literature values for bulk doping (Fig. 6). For the naphthalenediimide-bithiophene copolymer N2200 we achieve an average conductivity of 1.0 × 10^−3^ S cm^−1^ matching the values reported in the literature.^29,30^ In the case of the glycolated fullerene PTEG-1 we obtained an average value of 1 S cm^−1^, consistent with the findings of Liu *et al.*^25,31^ Notably, the method also proved effective for the ladder polymer polybenzimidazobenzophenanthroline (BBL), a material known for its challenging processability.^32^ Here, we obtained an average conductivity of 0.23 S cm^−1^. Although the value is lower than that achieved by the solution-based sequential doping method developed by Wang *et al.*,^17^ the processing parameters used for these materials were identical to those optimized for PCBM. No additional material-specific optimization was performed, underscoring the robustness and adaptability of our approach.

**Fig. 7:**
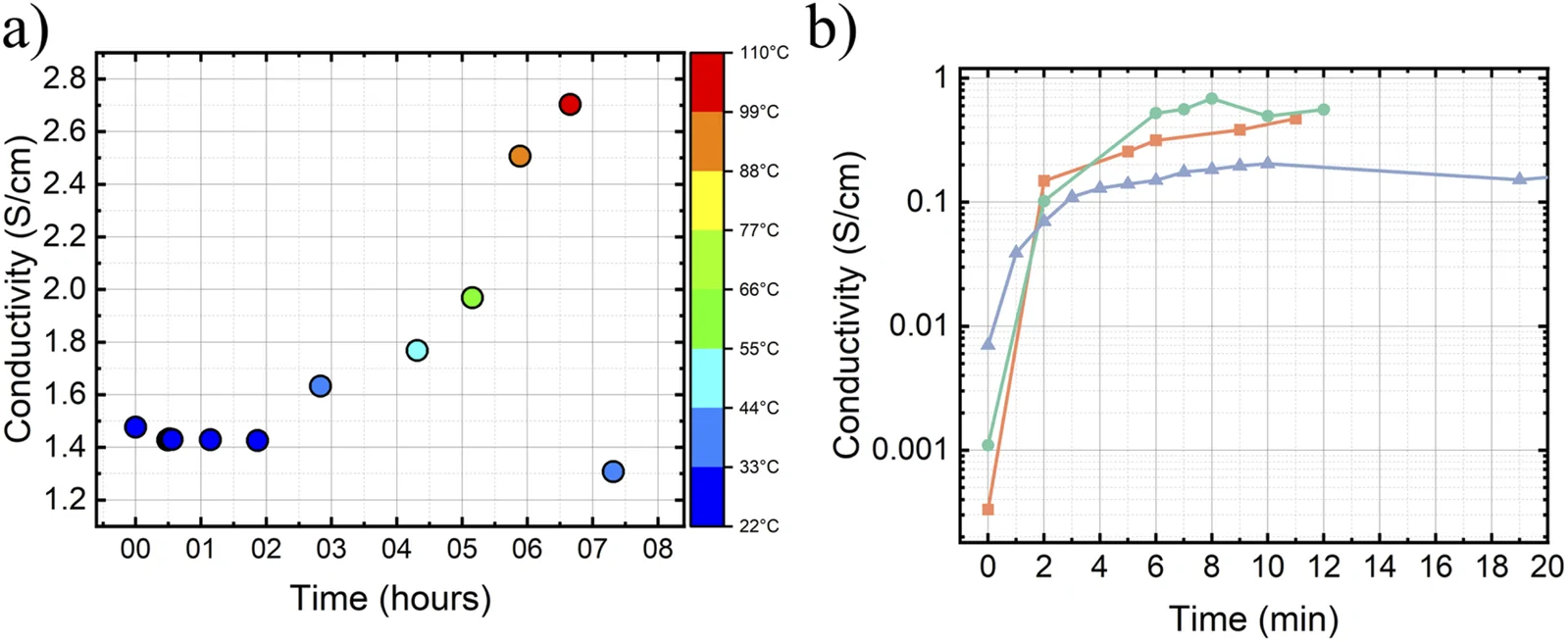
(a) Conductivity of an encapsulated and vapor-doped PCBM sample measured in ambient air at different temperatures. (b) Conductivity evolution of encapsulated PCBM after vapor doping treatment upon annealing in air at 120 °C.

A persistent challenge for n-type organic semiconductors is their poor air stability. To address this issue, we investigated whether encapsulation could mitigate degradation. We began by vapor-doping a PCBM sample and annealing it in a nitrogen environment. The sample was then encapsulated by bonding a glass slide onto the semiconductor film using a two-component epoxy glue, after which it was exposed to ambient air. Notably, the conductivity remained unchanged, prompting us to assess its stability at moderate temperatures. When the sample was heated to 120 °C, the conductivity decreased by only ∼15% after the thermal cycle, indicating a promising thermal stability (see Fig. 7a).

The deposition of N-DMBI-H is performed by heating the source film at relatively low temperatures—critically, below the onset temperature at which N-DMBI-H reacts with molecular oxygen.^31^ This observation led us to explore the air compatibility of the vapor-doping process. However, initial attempts to complete all processing steps in air yielded inconsistent results, likely due to residual oxygen exposure during post-doping handling. Consequently, we adopted a hybrid approach: samples were transferred to a glove box only for the encapsulation step before returning to ambient air for annealing and conductivity measurements. As shown in Fig. 7b, this method proved successful, achieving conductivities up to 0.7 S cm^−1^.

While these values are lower than those achieved with samples processed entirely in an inert atmosphere, they remain impressive given that only the encapsulation step was performed under nitrogen. This method thus offers a promising solution to the air instability that affects most n-type organic semiconductors.

## Conclusions

We demonstrated n-doping of organic semiconductors *via* vapor deposition using the commercially available N-DMBI-H without the need for specialized vacuum systems or the use of environmentally harmful solvents. The process is optimized for the methanofullerene PCBM yielding a remarkably high conductivity above 1 S cm^−1^. Vapor doping is then performed onto three more organic semiconductors demonstrating the generality of the approach. Finally, we demonstrated that the deposition process can be carried out in air with only minor drops in performance. This has implications for processing given that it allows to perform only one step in inert atmosphere, significantly reducing the pipeline complexity, cost and environmental impact.

## Materials and methods

### Material preparation and thin film deposition

PCBM, N-DMBI-H, N2200 (TCI), and PTEG-1 (Solenne), were dissolved in anhydrous chloroform (Extra Dry, Sigma-Aldrich) at a concentration of 10 g L^−1^. BBL (Sigma-Aldrich) was dissolved in methanesulfonic acid at a concentration of g L^−1^. Bulk doped N2200: dissolved at 5 g L^−1^ in CF mixed with a solution 10 g L^−1^ at a volume ratio of 100 : 3.

All films were deposited on borosilicate glass cleaned in an ultrasonic bath of soapy water, Milli-Q water, acetone, and isopropanol for 10 minutes for each solvent. Subsequently the substrates were dried and activated either *via* UV-O3 or O2 plasma right before being coated. N-DMBI-H source films were deposited *via* spin-casting at 300 rpm. N2200, PCBM, PTEG-1 films were deposited *via* spin-casting at 800 rpm. Bulk doping of PCBM and PTEG-1 was performed by mixing the semiconductor solution with the N-DMBI-H solution in a volume ratio of 10 : 1. BBL was spin cast at rpm at 1000 rpm immediately followed by a wash in isopropanol to remove the methanesulfonic acid. The BBL films were then dried on a hot plate at 80 C. For the ‘wet’ sequential doped BBL the N-DMBI-H solution was spin cast at 1500 rpm on top of the BBL film.

The semiconductor film was then placed above the N-DMBI-H source film separated by a controlled distance. This assembly was enclosed within a Petri dish to create a semi-sealed environment. The dopant vapor deposition was initiated by heating the N-DMBI-H source film on a hotplate to 85 °C (unless specified otherwise) for a controlled duration. (Note: vaporization of the N-DMBI-H was visible as an opaque white deposit formed on the cooler surfaces of the Petri dish.) Following the doping process, the semiconductor film was annealed at 120 °C, unless specified otherwise.

### Characterization

The resistance of the doped thin films prepared for the vapor deposition optimization study was measured in a N_2_-filled glove box with a Keithley 4200 SCS in a 4-point-probe configuration. The electrodes were patterned by evaporating through a shadow mask a thin layer of chromium (3 nm) followed by a layer of gold (25 nm). In four parallel strips of 6 mm width (*w*) and separated by 0.5 mm (*l*). Given the absence of a significant contact resistance between the gold electrodes and the doped PCBM films we switched to a 2-point-probe configuration for and measured the resistance with a Keithley 2700 in a probe station developed in-house (SI Fig. S4). To analyse the spatial distribution and homogeneity of the doping process, a larger substrate of 30 mm × 30 mm was chosen and the geometry of the electrodes modified to provide an array of 11 electrode pairs (see SI Fig. S3).

The conductivity was then calculated as:
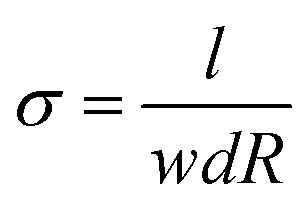
where *R* is the measured resistance and *d* is the thickness of the film. The thicknesses of the various films were measured either by AFM (Dimension Icon, Brucker) or with a stylus profilometer (Dektak, Bruker).

#### Quartz crystal microbalance

AT quartz crystal from McVac Inc., the resonance frequency of the crystal was then monitored with INFICON SQC-310 deposition controller. The shift in frequence was then converted into deposited mass using the Surbrey equation:
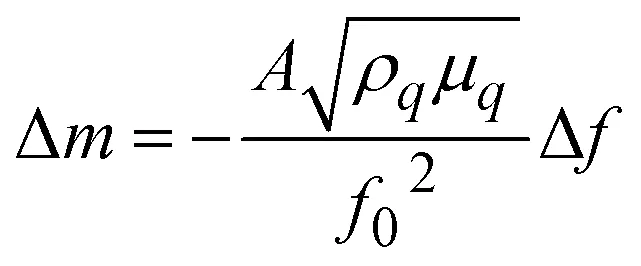
where *A* is the crystal area, *ρ*_q_ and *µ*_q_ are the density and shear modulus of the crystal, respectively, and *f*_0_ is the resonant frequency of the crystal.

## Author contributions

F. Ferrari: data curation, methodology, formal analysis, writing – original draft; O. Pokherniz: data curation, methodology, formal analysis; L. J. A. Koster: project administration, conceptualization, funding acquisition, writing – review & editing.

## Conflicts of interest

The authors declare no competing financial interests.

## Supplementary Material

RA-OLF-D6RA05776D-s001

## Data Availability

Data for this article, including raw IV curves are available at open science framework (OSF) at https://doi.org/10.17605/OSF.IO/ZU2P8. Supplementary information (SI) is available. See DOI: https://doi.org/10.1039/d6ra05776d.
