# Supplementary material for: Low-temperature, vacuum-free deposition of N-DMBI-H enables high conductivities in organic semiconductors
Source: RSC Adv. 2026 Aug 28;16(42):51099–105. doi: 10.1039/d6ra05776d (PMC13523653; doi:10.1039/d6ra05776d)
Supplement: Supplementary file 1 [file RA-016-D6RA05776D-s001.pdf]

Supplementary Information (SI) for RSC Advances.
This journal is © The Royal Society of Chemistry 2026

Supporting information

Low-temperature, vacuum-free deposition of N-DMBI-H enables high conductivities in organic semiconductors

Federico Ferrari[1], Oliver Pokerznik[1], L. Jan Anton Koster[1]

1 Zernike Institute for Advanced Materials, University of Groningen, Nijenborgh 3, 9747 AG, Groningen, The Netherlands

Table S 1: Conductivity and process parameters reported for n-type vapor doping of fullerenes

Host	Dopant	Pressure	Deposition Temperature (°C)	Conductivity (S/cm)	Ref
C_{60}	Leuco Crystal Violet	4×10^{-4} Pa	Not reported	1.3×10^{-2}	[1]
C_{60}	Acridine orange	10^{-7} mBar	Not reported	4.9×10^{-2}	[2]
C_{60}	$W2(hpp)_4$	10^{-4} Pa	Not reported	4	[3]
C_{60}	o-MeO-DMBI-I	10^{-7} mBar	Not reported	5.5	[4]
C_{60}	Cs_2CO_3	5×10^{-4} Pa	600	8.6	[5]
PCBM	PEI	1 atm	RT	5×10^{-4}	[6]
PCBM	TDAE	1 atm	RT	10^{-2}	This work
PCBM	N-DMBI-H	1 atm (in air)	RT	1	This work

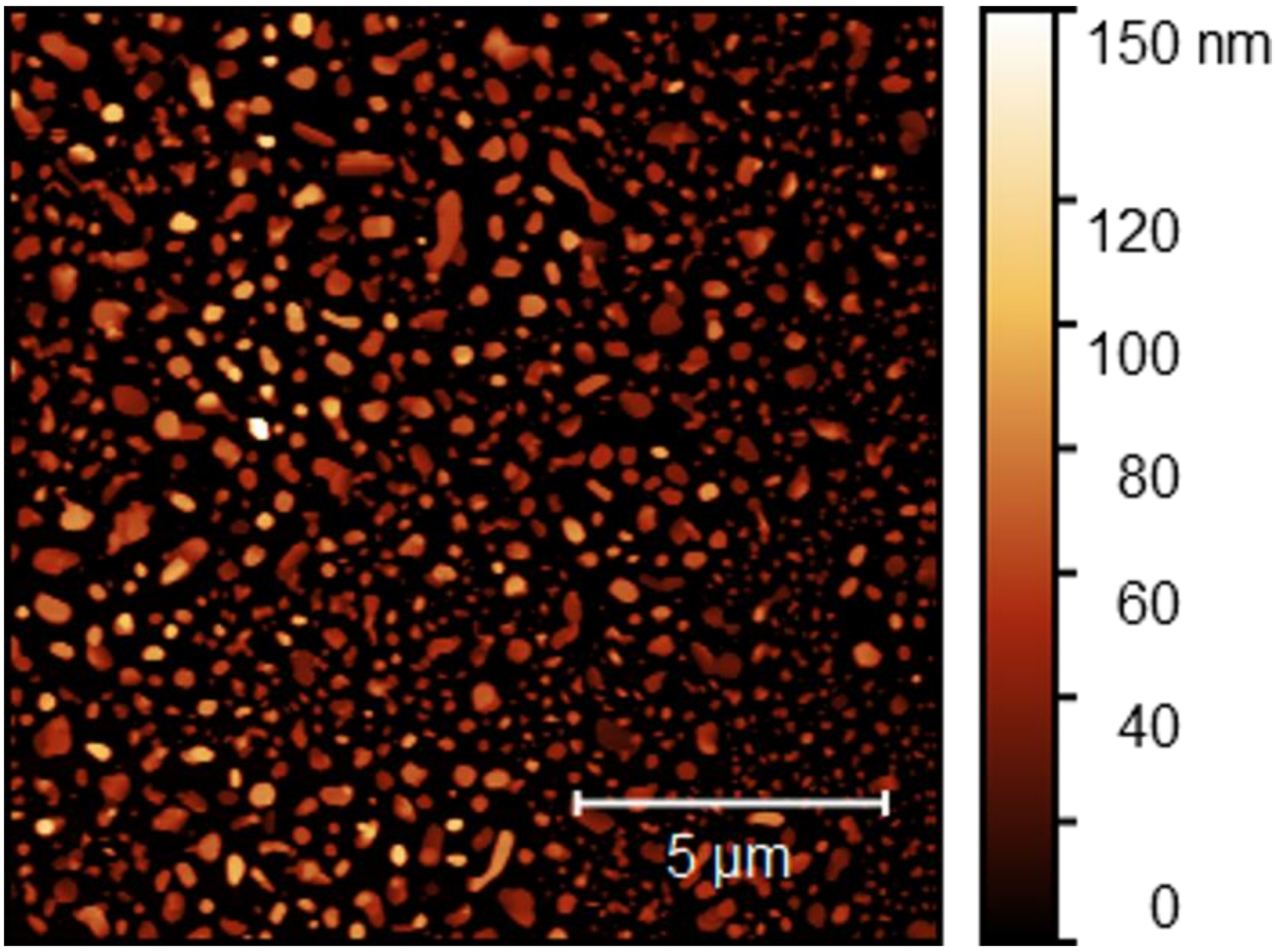

Figure S1: AFM height image of a glass slide exposed to N-DMBI-H vapor at 85°C for 25 minutes.

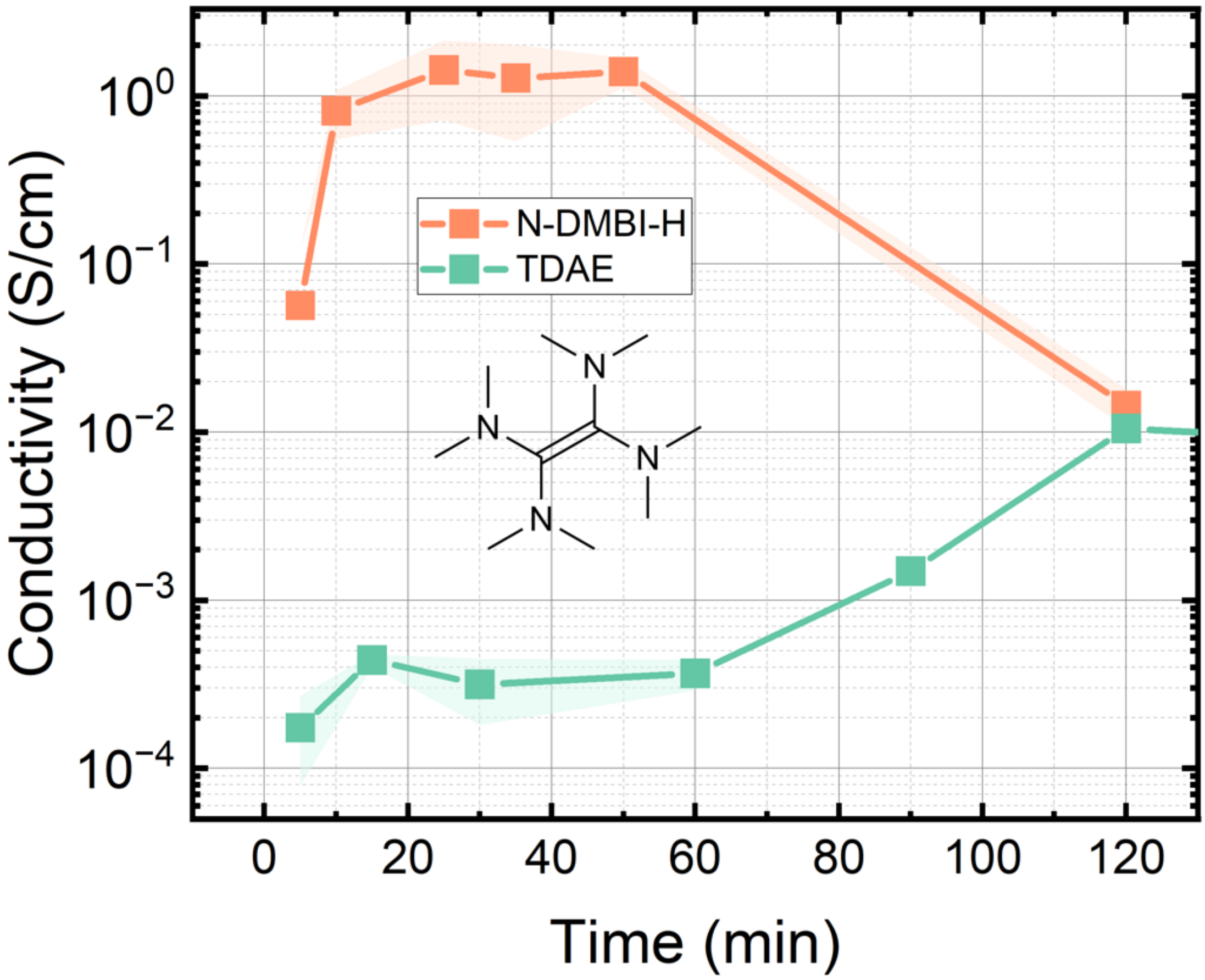

Figure S2: a) Conductivity of PCBM films upon exposure to the vapour of tetrakis(dimethylamino)ethylene (TDAE) and N-DMBI-H for different durations. The molecular structure of TDAE is shown in the graph.

The conductivities obtained upon exposure of TDAE reach 10^{-2} S/cm. To the best of our knowledge there are no reports of PCBM doped with TDAE, only C60-TDAE systems were reported to have conductivities only up to 10^{-4} S/cm.

When PCBM is exposed to TDAE, the resulting conductivities reach up to 10^{-2} S/cm. While these values are significantly lower than PCBM vapor doped with N-DMBI-H, this stands in stark contrast to previous reports: to the best of our knowledge, only C_{60}-TDAE charge-transfer crystals have been documented, and even those achieved conductivities no higher than 10^{-4} S/cm.[7–9]

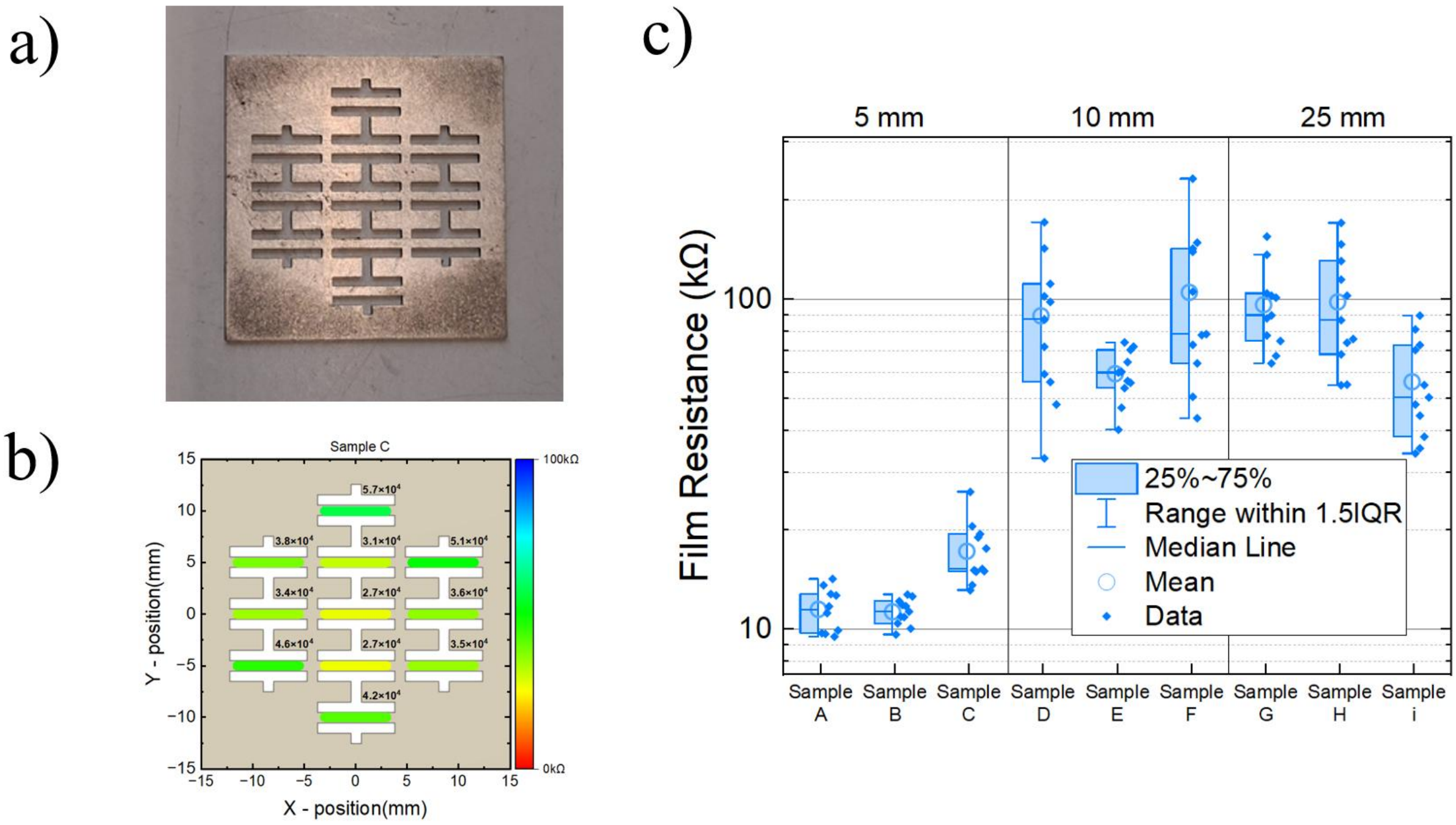

Figure S3: a) Shadow masks used for patterning of the electrodes used to check the spatial homogeneity of the vapor doping process. b) Example of a resistances measured at different location on a substrate (values reported in Ω). c) Boxplot showing resistance variability within and across samples.

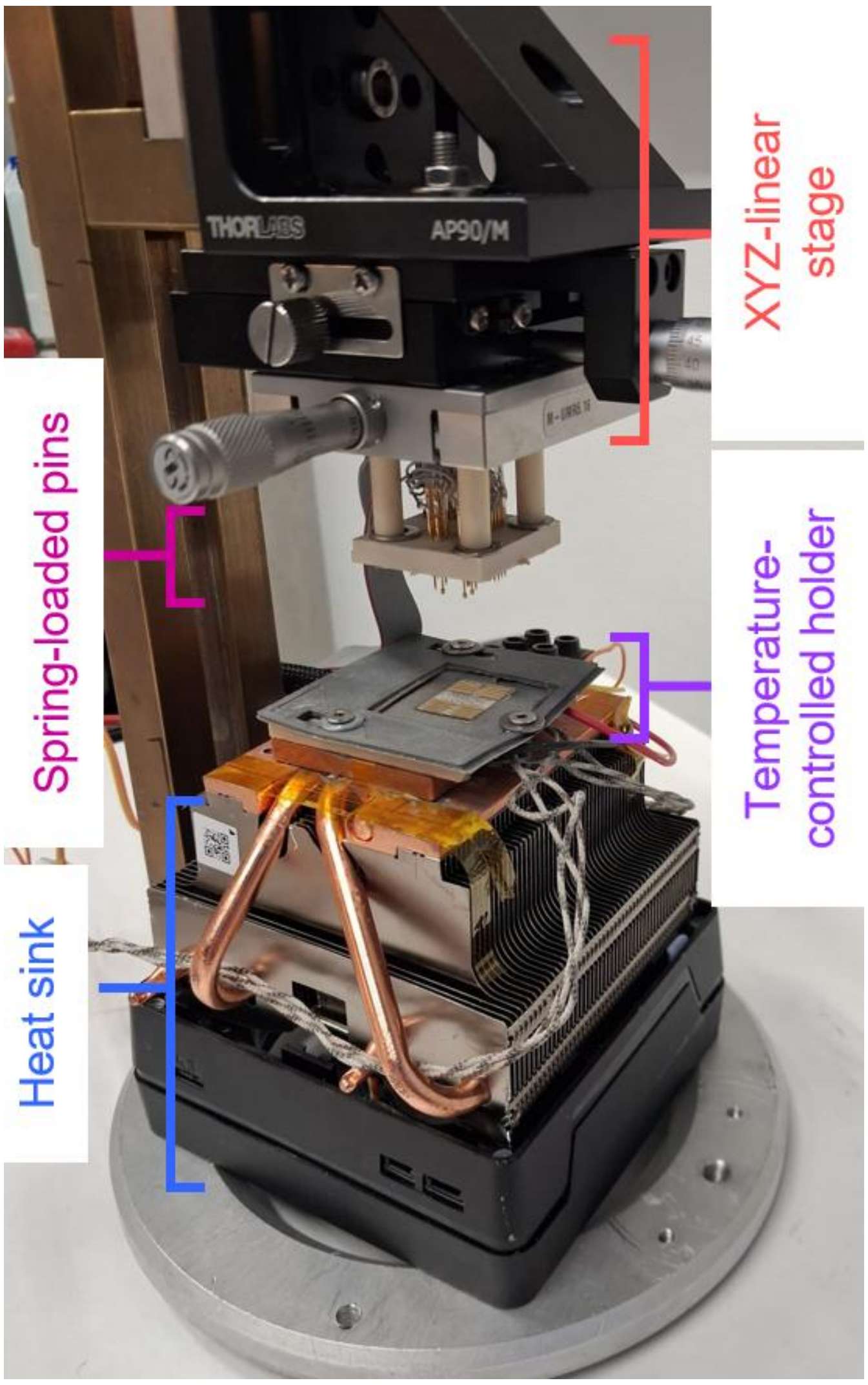

Figure S4: Setup used for the measurement of the resistance during the annealing.

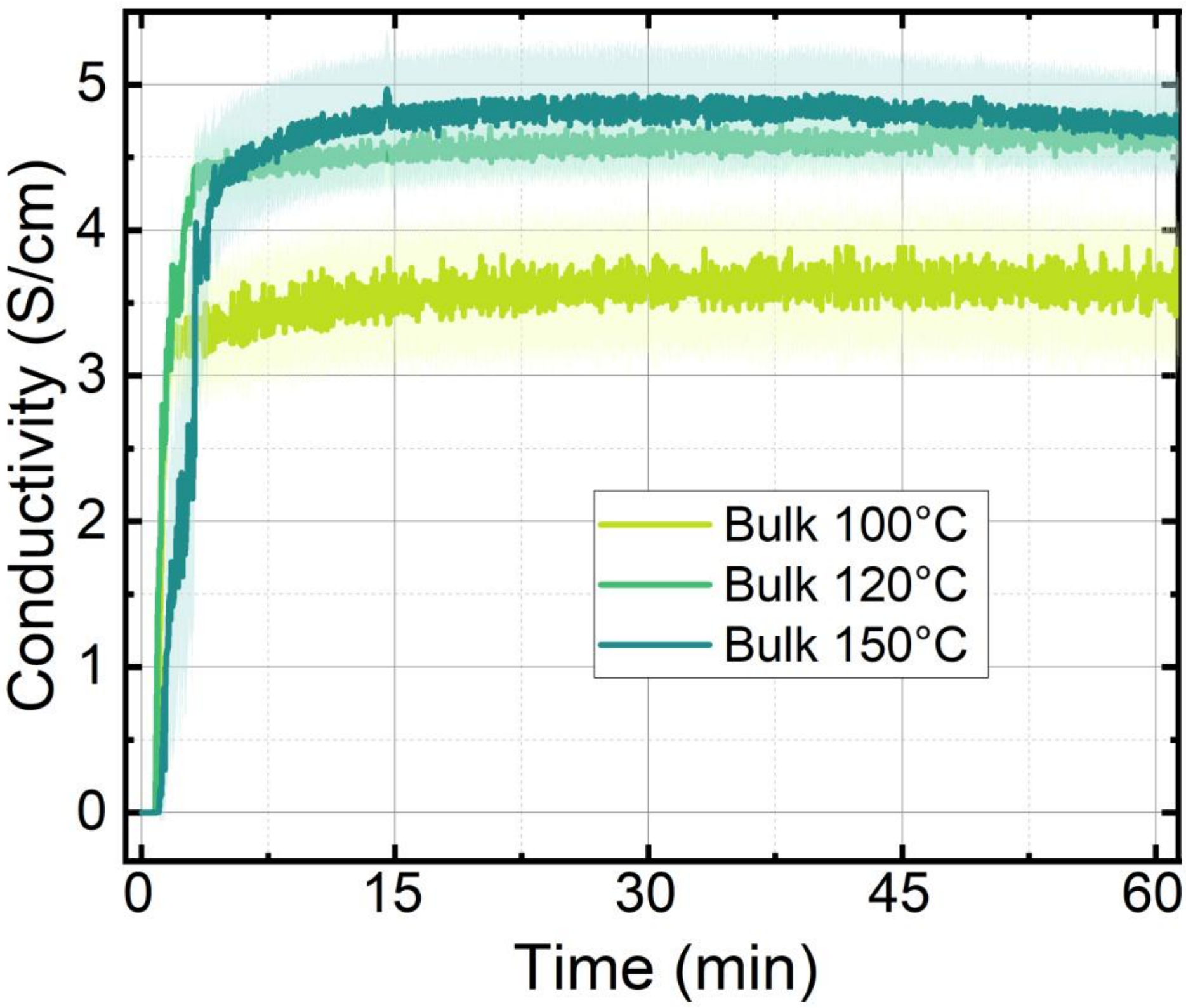

Figure S5: Average trends in conductivity of multiple measurements (at least 12 from three different samples) during the annealing at three different temperatures for bulk doped PCBM.

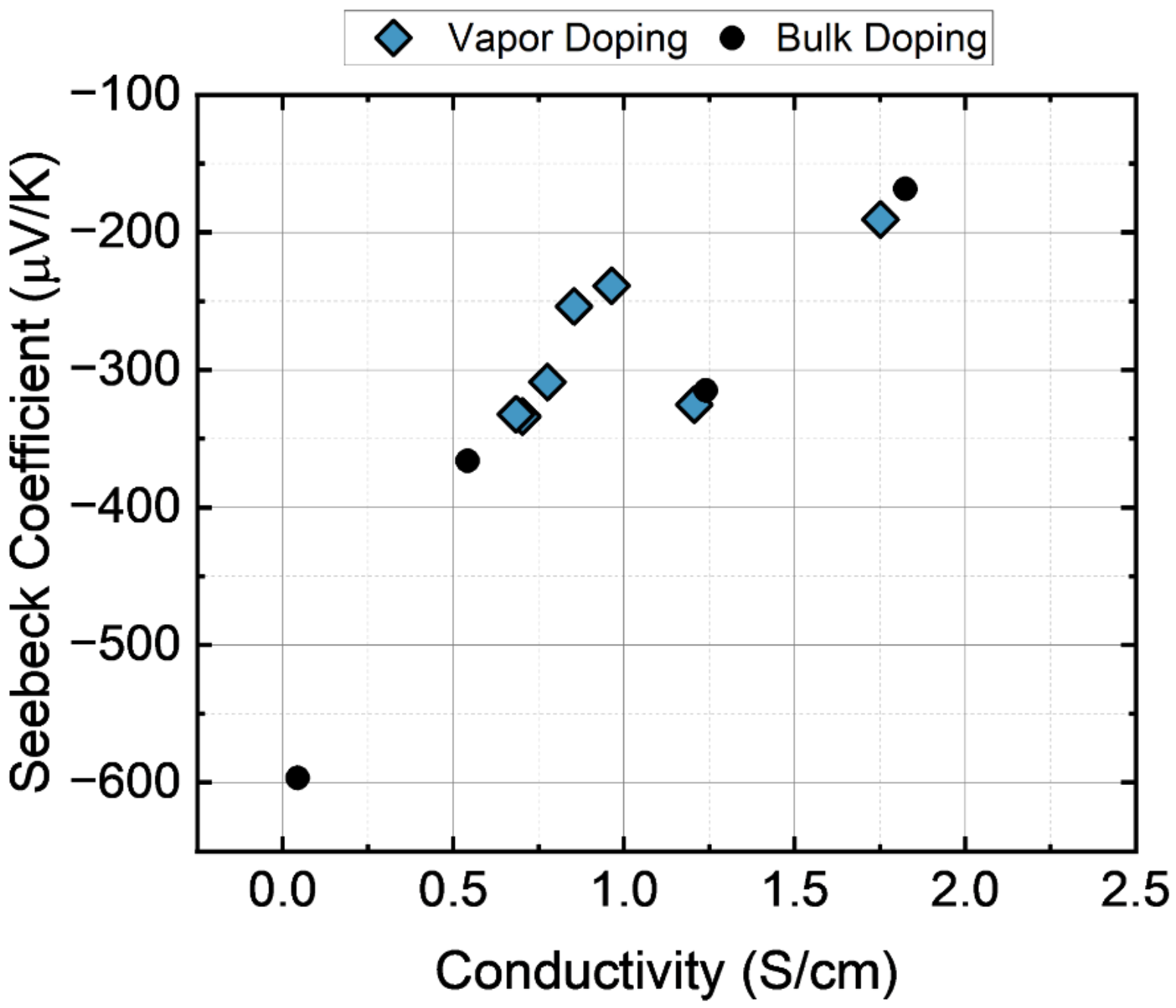

Figure S6: Measured Seebeck coefficient for vapor doped (blue diamonds) and bulk doped (black dots) PCBM at different doping levels. Given the alignment of the two datasets we consider the two doping methodologies to yield equivalent materials.

[1] F. Li, A. Werner, M. Pfeiffer, K. Leo, X. Liu, *Journal of Physical Chemistry B* **2004**, *108*, 17076.

[2] F. Li, M. Pfeiffer, A. Werner, K. Harada, K. Leo, N. Hayashi, K. Seki, X. Liu, X. D. Dang, *J. Appl. Phys.* **2006**, *100*.

[3] T. Menke, D. Ray, J. Meiss, K. Leo, M. Riede, *Appl. Phys. Lett.* **2012**, *100*.

[4] P. Wei, T. Menke, B. D. Naab, K. Leo, M. Riede, Z. Bao, *J. Am. Chem. Soc.* **2012**, *134*, 3999.

[5] M. Sumino, K. Harada, M. Ikeda, S. Tanaka, K. Miyazaki, C. Adachi, *Appl. Phys. Lett.* **2011**, *99*.

[6] S. Fabiano, S. Braun, X. Liu, E. Weverberghs, P. Gerbaux, M. Fahlman, M. Berggren, X. Crispin, *Advanced Materials* **2014**, *26*, 6000.

[7] A. Omerzu, D. Mihailovic, N. Biskup, O. Milat, S. Tomic, *Synth. Met.* **1997**, *85*, 1723.

[8] A. Omerzu, D. Mihailovic, S. Tomic, O. Milat, N. Biskup, **1996**.

[9] A. Schilder, H. Klos, I. Rystau, W. Schiitz, B. Gotschy, *REVIEW LETTERS* **1994**, *73*.
